# Characterization and Safety Profile of a New Combined Advanced Therapeutic Medical Product Platelet Lysate-Based Fibrin Hydrogel for Mesenchymal Stromal Cell Local Delivery in Regenerative Medicine

**DOI:** 10.3390/ijms24032206

**Published:** 2023-01-22

**Authors:** Thibault Canceill, Géraldine Jourdan, Philippe Kémoun, Christophe Guissard, Yanad Abou Monsef, Marion Bourdens, Benoit Chaput, Sandrine Cavalie, Louis Casteilla, Valérie Planat-Bénard, Paul Monsarrat, Isabelle Raymond-Letron

**Affiliations:** 1 CIRIMAT, Université Toulouse III Paul Sabatier, CNRS UMR 5085, INPT, Faculté de Pharmacie, 35 Chemin des Maraichers, CEDEX 09, 31062 Toulouse, France; 2 Department of Oral Medicine and Toulouse University Hospital (CHU of Toulouse)—Toulouse Institute of Oral Medicine and Science, CEDEX 09, 31062 Toulouse, France; 3 RESTORE Research Center, Université de Toulouse, INSERM, CNRS, EFS, ENVT, Batiment INCERE, 4bis Avenue Hubert Curien, 31100 Toulouse, France; 4 LabHPEC, Histology and Pathology Department, Université de Toulouse, ENVT, CEDEX 03, 31076 Toulouse, France; 5 Service de Chirurgie Plastique, Reconstructrice et Esthétique, Centre Hospitalier Universitaire Rangueil, Avenue du Professeur Jean Poulhès, CEDEX 09, 31059 Toulouse, France; 6 Artificial and Natural Intelligence Toulouse Institute ANITI, 31400 Toulouse, France

**Keywords:** regenerative medicine, adipose-derived mesenchymal stromal cells, platelet–lysate, fibrin hydrogel, preclinical safety, biodistribution

## Abstract

Adipose-derived mesenchymal stromal cells (ASC) transplant to recover the optimal tissue structure/function relationship is a promising strategy to regenerate tissue lesions. Because filling local tissue defects by injection alone is often challenging, designing adequate cell carriers with suitable characteristics is critical for in situ ASC delivery. The aim of this study was to optimize the generation phase of a platelet–lysate-based fibrin hydrogel (PLFH) as a proper carrier for in situ ASC implantation and (1) to investigate in vitro PLFH biomechanical properties, cell viability, proliferation and migration sustainability, and (2) to comprehensively assess the local in vivo PLFH/ASC safety profile (local tolerance, ASC fate, biodistribution and toxicity). We first defined the experimental conditions to enhance physicochemical properties and microscopic features of PLFH as an adequate ASC vehicle. When ASC were mixed with PLFH, in vitro assays exhibited hydrogel supporting cell migration, viability and proliferation. In vivo local subcutaneous and subgingival PLFH/ASC administration in nude mice allowed us to generate biosafety data, including biodegradability, tolerance, ASC fate and engraftment, and the absence of biodistribution and toxicity to non-target tissues. Our data strongly suggest that this novel combined ATMP for in situ administration is safe with an efficient local ASC engraftment, supporting the further development for human clinical cell therapy.

## 1. Introduction

Regenerative therapies using natural or synthetic materials to promote long-lasting tissue structuration and reconstruction remain a challenge, as they still provide very unpredictable and only partial regeneration outcomes [1,2,3,4]. When associated with biomaterials, mesenchymal stromal cells (MSC) implantation increases the biotherapy procedure efficacy [5,6]. Indeed, in situ administration of MSC is likely to induce a deep and sustainable modification of the microenvironment for tissue regeneration [7]. Although some of the therapeutic effects may be supported by MSC multipotency, it is rather admitted that MSC exert strong features mediated by their pleiotropic paracrine activity, among which they have the ability to regulate both angiogenesis and immunity [8] and to exert anti-infectious activity [9]. MSC also largely condition tissue architecture through their constant remodeling of extracellular matrix (ECM) supporting the mechanical macrostructure of any tissue [10]. Poor survival of administered MSC in damaged areas may limit therapy effectiveness. Indeed, a hostile microenvironment for MSC engraftment, with insufficient vascular supply, inadequate tissue factors or unfavorable biomechanics may compromise the therapy outcomes. Therefore, several studies demonstrated the lack of therapeutic benefit, including tissue regeneration, by using MSC in an improper vehicle [11,12]. Conversely, it is reported that the MSC delivery in an appropriate biomaterial carrier can exert its effects at several levels, including fibrin clot maintenance resulting from post-surgical coagulation, neovascularization, immunomodulation and the recruitment of the endogenous progenitors leading to tissue regeneration [13,14,15,16].

Among the cellular carriers proposed in regenerative medicine, platelet-derived products and especially carriers based on platelet–lysate (PL) have recently received particular attention. PL refers to the content of the platelet cytoplasm released after membrane disruption and that can be obtained from plasma platelet concentrates. Indeed, benefiting from both plasma and platelet content, such products are strongly enriched in cytokines/growth factors (e.g., VEGF (Vascular Endothelial Growth Factor), PDGF (Platelet-Derived Growth Factor), EGF (Epidermal Growth Factor) or TGF-β (Transforming Growth Factor-β)), fibrinogen (concentrations up to 3 mg/mL) along with other proteins and ions [17,18]. Human PL is already provided as a commercial validated product for cell culture (meeting the requirements of Good Manufacturing Practices, i.e., GMP) and as an alternative to bovine serum [19,20,21]. In addition, fibrinogen contained in PL provides a full option to obtain a biodegradable scaffold. Indeed, by polymerization of fibrinogen contained into PL, a fibrin hydrogel enriched in growth factors can be obtained, mimicking an extracellular matrix [22]. These scaffolds can be progressively degraded by plasmin and matrix metalloproteinases (MMPs), resulting in full biodegradability and host integration. For in vivo implantation, PL-based fibrin hydrogels (PLFH) would provide a favorable environment for the survival and development of therapeutic cells [23]. This type of matrix is described as a “temporary natural matrix” that is intended to be replaced by a new natural extracellular matrix once the implanted network is resorbed [24]. However, although fibrin hydrogel has been reported to optimize cell therapy, little is known about the suitability of PLFH as an MSC regenerative medicine carrier, including those from adipose tissue (ASC). Indeed, for many reasons related to safety in tissue donor processing, tissue availability and therapeutic activities, ASC are expected to be a valuable source of cells and are being increasingly tested at a clinical level [25]. In addition, when delivered in the PL-based carrier, exogenous ASC therapy appears to be a promising strategy to promote complex, composite soft/mineralized tissue regeneration, such as joint or tooth supporting tissues (i.e., periodontium) [26].

Such an association of a carrier hydrogel and ASC are classified by the European Medicines Agency (i.e., EMA [27]) as a combined Advanced Therapy Medicinal Product (i.e., ATMP). Although the vast majority of clinical trials using MSC derived from various sources has not revealed any significant treatment-related health concerns [28], combined ATMP may carry specific risks. Potential adverse events could arise from the cells or the carriers, such as localized host tissue response, differentiation into unwanted cell types, long-term persistence or unscheduled biodistribution. To be used as an ATMP in human or animal medicine (“one medicine”), tissue response after PLFH/ASC delivery in vivo implantation including final integration or absorption/degradation of the material is mandatory. Such risks are evaluated in preclinical animal model studies as part of a comprehensive safety program prior to administration in human.

Thus, the aim of this study was to optimize the generation step of the PLFH/ASC ATMP for the purpose of medical use and (1) to investigate in vitro PLFH biomechanical properties, cell viability, proliferation and migration sustain, and (2) to assess in vivo PLFH/ASC biosafety: tolerance after the local grafting, biodegradability of the PLFH carrier, ASC fate (tissues integration, potential long term persistence and proliferation) and finally biodistribution and toxicity of the cells (study of the non-target tissues).

## 2. Results

### 2.1. Polymerization Dynamics of PLFH Hydrogels

**Figure 1:**
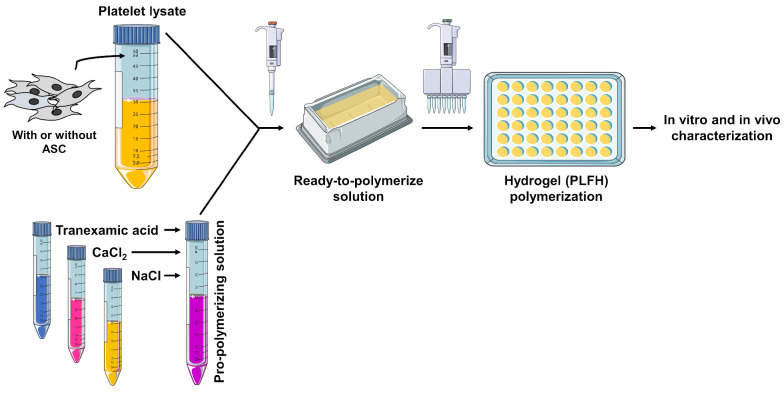
Schematic representation of PLFH preparation.

The process for obtaining PLFH is summarized in Figure 1.

**Figure 2:**
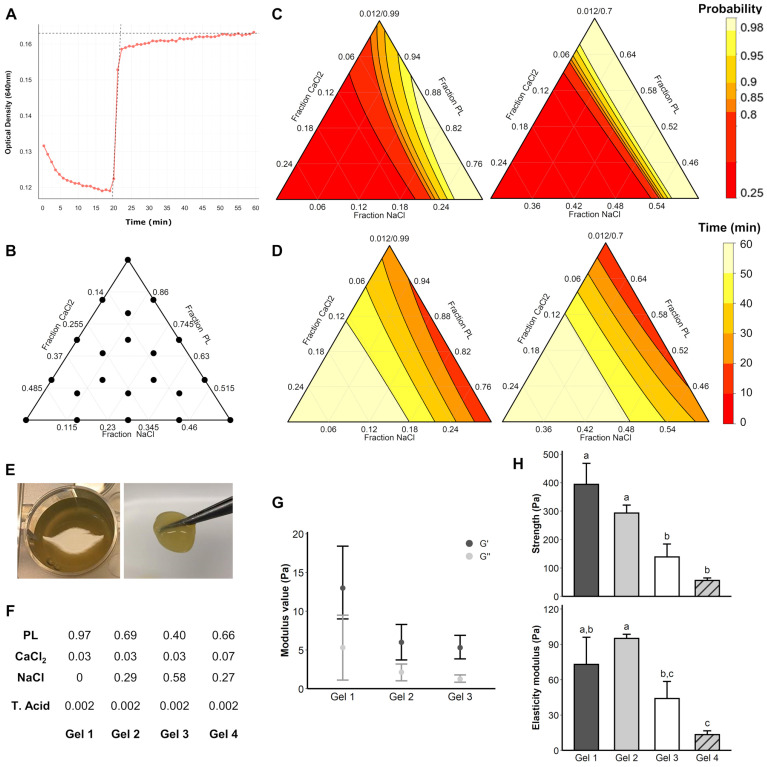
PLFH polymerization assessment, rheology and surface resistance. (**A**) Example of polymerization curve obtained by monitoring the optical density OD at 640 nm. A sharp increase in OD was observed at the time of polymerization. The polymerization time was defined as the time at the intersection of the tangent to the exponential growth phase with the asymptote (here: t = 22 min). (**B**) Mixture experiment highlighting the proportions to be tested for each hydrogel component. (**C**) Probability modeling of obtaining polymerization of hydrogels after one hour at 37 °C as a function of the different proportions of the constituents. The reddest areas correspond to the lowest probabilities. (**D**) Polymerization time modeling as a function of the different proportions of the constituents. The reddest areas show the fastest polymerizations. (**E**) Photograph of a slightly translucent polymerized hydrogel adhering to the walls and a hydrogel caught with forceps. (**F**) Gel 1 to 4 mix component amounts. (**G**) Mean storage modulus (G’) and loss modulus (G”) calculated at 0.5% strain over a frequency sweep from 0.01 to 1 Hz (expressed as the mean ± standard deviation). (**H**) Mechanical wear of the PLFH under compressive stress at a crosshead speed of 2 mm/min. An identical letter indicates non-significant differences.

Briefly, a pro-polymerizing solution containing NaCl, $CaCl_{2}$ and tranexamic acid is added to platelet–lysate for subsequent polymerization. Optical density monitoring highlighted the hydrogel formation dynamics, with a sudden optical density increase at the beginning of the gelation process (Figure 2A). In order to explore how the variation of proportion of each component influences the dynamics of hydrogel polymerization, a “mixture experiment” methodology was followed to explore the space of parameters, resulting in 21 different mixes to test (Figure 2B). $CaCl_{2}$ and PL variation had the strongest impact on polymerization probability (Figure 2C), leaving the amount of NaCl as an adjustment factor to remain at constant volume. Increasing the $CaCl_{2}$ amount resulted in a decrease in the polymerization probability (Figure 2C) and an increase of the polymerization time (Figure 2D), showing an optimal proportion below 8%. Using over 90% of PL in the final mix decreased the polymerization probability (Figure 2C). The 0.4–0.97% PL range displaying the optimal proportions both to increase the polymerization probability and to remain in acceptable polymerization time (Figure 2C,D), is therefore compatible with a future in vivo and clinical protocol use. Figure 2E shows the appearance of the gel once polymerized and after removal from culture well. Finally, from this broad pre-screening step, four gel mixes (i.e., “Gel” 1 to 4, Figure 2F) were selected for further mechanical characterization.

### 2.2. Characterization of PLFH Mechanical Properties

The rheometer frequency sweep indicated that for the four conditions tested, the G’ modulus was greater than the G” modulus, confirming the rheological solid state of the structure and therefore the possibility of qualifying it as a gel (Figure 2G). The compression on the surface of the PLFH required a stress, which was more important, as the amount of PL was increased (Figure 2H). No statistically significant differences were found between the Gel 1 and 2, and between the Gel 3 and 4 regarding mechanical characteristics. In conclusion, Gel 1 and 2 have better mechanical characteristics than Gel 3 and 4, with the highest elastic modulus observed for Gel 2 (Figure 2H).

### 2.3. Physicochemical Properties of PLFH

**Figure 3:**
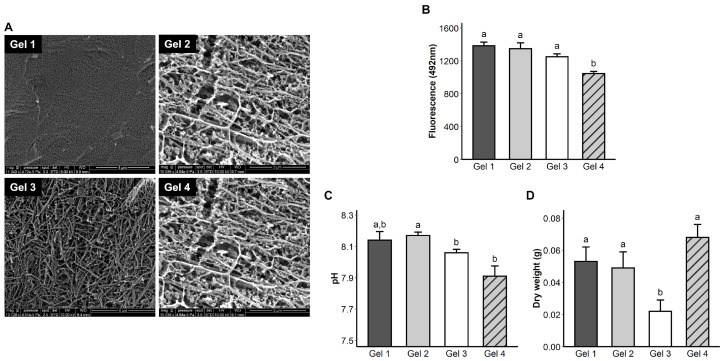
Hydrogels’ physicochemical properties. (**A**) Observation of the fibrin network by electron microscopy. Gel 1: very dense fiber mesh, smaller pores. Gel 2: looser mesh, more porous with longer fibers. Gel 3: much less tight mesh, more porous network. Gel 4: intermediate mesh made of shorter fibers. (**B**) Determination of the fluorescence in the supernatant of the hydrogels, after deposition of a 0.5 mg/mL solution of 70 kDa fluorescein-dextran left for 24 h. Lowest fluorescence means highest diffusion and porosity. The porosity of the gel, allowing the diffusion of these molecules, was influenced by the amount of $CaCl_{2}$ and PL. (**C**) pH measurement after their constitution. (**D**) PLFH weights after drying on a pad (mass of the matrix network). An identical letter indicates non-significant differences.

The Cryo-MEB investigation on PLFH demonstrated that the fibrin network organization depended on mix type. Gel mix with the greatest PL amount displayed the highest fibrin network meshes density (Figure 3A, Gel 1), whereas the spaces between the fibers were wider, opening more pores, when this PL proportion was low (Gel 2 versus 3, Figure 3A). With high $CaCl_{2}$ amounts, the fibers were shorter than in other conditions (Figure 3A, Gel 4 versus Gels 1, 2 and 3). Hydrogels with a high $CaCl_{2}$ and low PL proportion significantly demonstrated faster diffusion of fluorescent 70 kDa dextran (Figure 3B), confirming that PLFH porosity increased with the $CaCl_{2}$ amount. PLFH showed a slightly basic pH (around pH 8). Gel 2 had the highest average pH value and significantly exhibited a more basic pH than Gel 3 and 4 (Figure 3C). Average PLFH wet weight was 714 ± 112 mg and 48 ± 23 mg after drying. PLFH lost on average 93 ± 4% of their weight after drying. The hydrogel with the lowest PL amount (Gel 3) significantly exhibited the lowest dry weight (Figure 3D).

### 2.4. PLFH Enhances ASC Migration and Proliferation In Vitro

**Figure 4:**
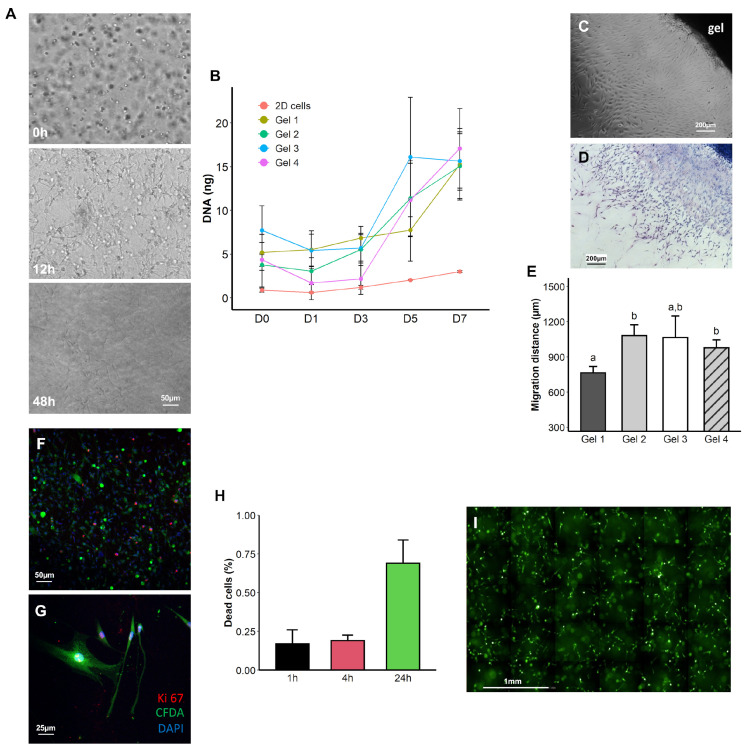
In vitro ASC features in PLFH. (**A**) Light microscopy visualization of ASC inside Gel 2 immediately after the formation of the PLFH biohybrid, at 12 and 48 h. Starting with a rounded shape when not yet adherent, ASC progressively acquired a "fibroblastic" star-like appearance, showing spreading and adhesion within the matrix network. (**B**) Evaluation of cell proliferation in the PLFH by DNA quantification. DNA amounts increased between day 3 and day 5. Cells left adherent on plastic were used as a control (2D cells). (**C**,**D**) Visualization at day 5 of ASC that have migrated out of the hydrogel (Gel 2), by light microscopy (**C**) and after fixation and staining with May-Grünwald Giemsa (**D**). (**E**) Evaluation of the average migration distance (in µm) by measuring the distance between the hydrogel and the migration front at day 5. Low (**F**) and high (**G**) magnification Ki-67 immunostaining of PLFH/ASC (Gel 2) showing proliferating ASC (red nuclei staining) 72 h after being seeded inside the hydrogel. CFDA and DAPI stained cell cytoplasm and nuclei, respectively. (**H**,**I**) CFDA/DAPI staining and Operetta® microscopy for assessment of ASC viability inside Gel 2. Quantification of cell viability 4 and 24h after cell seeding inside PLFH displays less than 1% of dead cells (DAPI positive, blue) from total ASC population. An identical letter indicates non-significant differences.

Whatever the PLFH component proportions, ASC displayed “fibroblastic” star-like appearance with spreading and adhesion 48 h after seeding within the gel (Figure 4A). For each PLFH mix, DNA amount increased from day 3 in similar amount (Figure 4B). The average doubling time in the PLFH was thus 4.4 days, similar to ASC doubling-time in 2D culture (4 days). ASC started to migrate out of the PLFH as early as day 3 of culture. Measurement of migration distances from gels’ edge (Figure 4C,D) on day 5 showed that cells from the Gel 1 (displaying the highest PL percentage) significantly migrated less than Gels 2 and 4 (Figure 4E). Numerous ASC exhibited Ki-67 nuclear staining 72 h after seeding in Gel 2, confirming their ability to proliferate (Figure 4F,G, Supplementary Figure S1). The cell viability experiment showed less than 1% of dead cells 24 h after ASC seeding in Gel 2 (Figure 4H,I).

In vitro experiments demonstrated than Gel 2, with 69% PL and 3% $CaCl_{2}$ having adequate mechanical properties (strengths and elastic modulus), suggesting easy handling and subsequent in vivo administration while maintaining cell viability, proliferation and migration.

### 2.5. Ergonomics and Macroscopic Findings

PLFH ergonomics (consistency and elasticity) for surgical handling was suitable for both subcutaneous and subgingival implantation site, malleable enough to fill a tissue defect (subgingival site) and strong enough to retain its shape over time despite progressive degradation (subcutaneous implantation). Macroscopic examination of flank subcutis grafted sites at necropsy demonstrated the local successful engraftment, the supportive angiogenesis development and the progressive shrinking and disappearance of the smooth whitish mass over time (Supplementary Figure S2).

### 2.6. Histopathological Analysis Demonstrated an Effective PLFH Biodegradability and Overall Good Tolerance

**Figure 5:**
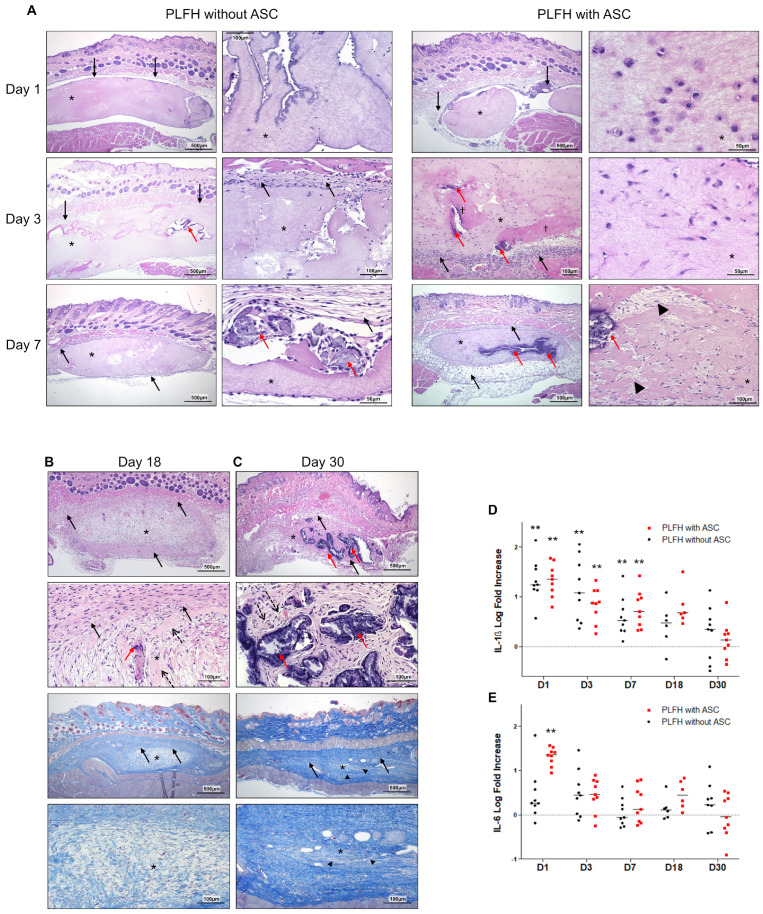

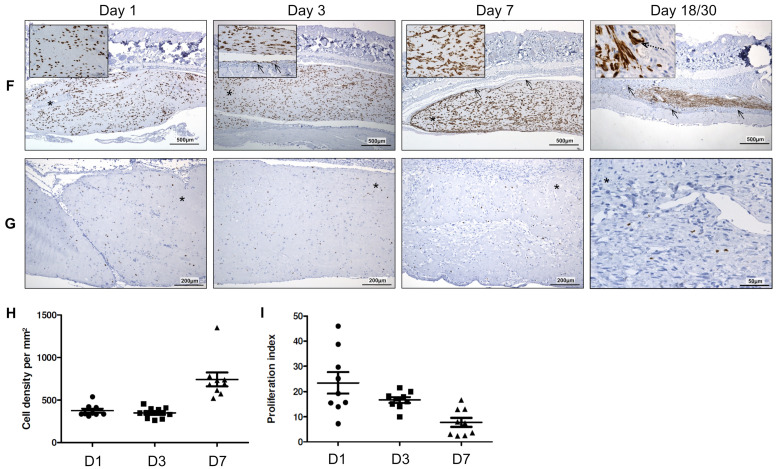
PLFH biodegradation, local tolerance and ASC fate after subcutaneous implantation. (**A**) Day 1, 3 and 7 after PLFH (*) implantation without (left panel) or with ASC (right panel), HE staining. Day 1 after implantation: homogeneous or focally stripped eosinophilic PLFH with a surrounding serous exudation (empty spaces) in the post-surgical pocket and a neutrophilic infiltration of both the PLFH and the murine recipients’ tissues. Minimal surrounding fibroblastic reaction was observed (black arrows). Day 3: mononuclear cells infiltrate with surrounding activated murine fibroblasts. Multifocal areas of calcium precipitates were observed (red arrows). Limited ASC death (ghost cells or empty spaces, †). Day 7: 10-15 layers of fibroblasts and collagen secretion (black arrows) surrounded PLFH. Prominent foreign body cell reaction developed around calcified areas (red arrows). Otherwise, ASC showed a typical networked fibroblast-like fusiform phenotype and secreted a clear extracellular matrix (black arrowheads). (**B**) Day 18 and (**C**) day 30 after PLFH (*) implantation with ASC (HE and Masson trichrome (MT) staining). (**B**) Day 18: dense collagenous reaction around PLFH (black arrows), and calcium precipitation with multinucleated giant cells (red arrows). Loose collagen fibers secreted by ASC and rare PLFH residual fragments (dashed arrows). (**C**) Day 30: dense collagenous matrix deposition with high ASC density and scarce residual PLFH fragments. Variable amounts of concentrated and fragmented calcium crystals (red arrows) delineated by foreign body giant cell granulomatous reaction, dense mature collagenous fibrosis and discrete angiogenesis (dotted arrows). Dense collagen fibers, secreted by ASC, replaced PLFH (MT, black arrowheads). IL-1β (**D**) and IL-6 (**E**) gene expression. Significant difference between PLFH with or without ASC at the 0.01 level (**). (**F**) ASC morphology and persistence (vimentine IHC with brown staining of human ASC cytoplasms). Day 1: large, round ASC. Day 3: ovoid or spindle shaped ASC. Some ASC were observed at PLFH periphery (arrows on the magnified image). Day 7: dense ASC network within the PLFH. Days 18 and 30: dense ASC bundles infiltrating the PLFH together with ASC around the PLFH (arrows). Examples of ASC in perivascular positions (dashed arrow, magnified picture), in connective tissue and underlying muscle fibers (arrows). (**G**) ASC Ki-67 nuclear labeling: the number of positive cells decreased until day 30. (**H**) Cell density increased between day 1 and 7. (**I**) Proliferation index decreased from 20% (day 1) to 5% (day 7). Nine animals by timepoint and by group were used, except for 18 days (6 animals by group).

**Figure 6:**
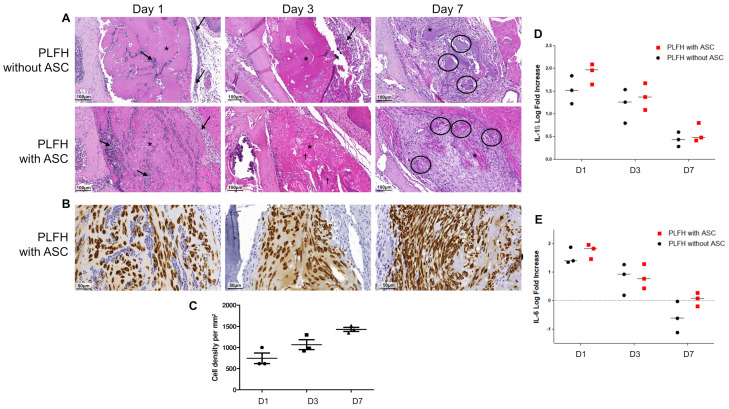
PLFH biodegradation, local tolerance and ASC fate after subgingival administration of miniaturized implant. (**A**) PLFH (*) biodegradation without (upper panel) or with ASC (middle panel) 1, 3, 7 days after surgical implantation. Day 1, neutrophilic infiltration (arrows) with a slight serous exudation around the PLFH (empty spaces). Day 3, PLFH fragmentation, resorption and shrinkage, areas of ASC death (†), peripheral mononuclear cell (mainly macrophages). Day 7, multifocal calcium precipitations with granulomatous foreign body reaction (circles) and significant PLFH resorption/biodegradation. (**B**) Vimentin immunostaining demonstrated the great ASC number, density, and viability. (**C**) Cell density doubled between day 1 and 7. (D-E) IL1-β (**D**) and IL6 (**E**) expression in subgingival tissue after PLFH with or without ASC implantation. Three animals by timepoint and by group were used.

Similar changes were observed after subcutaneous (Figure 5) or subgingival implantation (Figure 6). Local evolution/resorption of PLFH was not influenced by the ASC presence. Microscopic evaluation at early timepoints showed homogeneous or focally stripped PLFH (day 1), moderate and diffuse neutrophilic infiltration and slight serous exudation, probably linked to the surgical procedure (Figure 5A and Figure 6A). From day 3 to 7, PLFH underwent progressive resorption, with calcium salt precipitations, fragmentation and shrinking. Inflammation switched from neutrophilic to mononuclear cells (mainly macrophages and lymphocytes infiltration). A moderate multifocal foreign body giant cell reaction, triggered by calcium precipitates (Figure 5A and Figure 6A), was often prominent. This granulomatous reaction decreased in a time dependent manner (Figure 5A–C and Figure 6A) with progressive fragmentation and biodegradation of the PLFH. The implanted site developed a peripheral light to moderate murine fibrosis reaction around the PLFH (Figure 5A and Figure 6A). At later timepoints (D18/30), PLFH were almost completely resorbed with remaining foreign body granulomatous reaction around the multifocal calcium deposits/precipitates (Figure 5B,C). Fibrosis was observed around the implanted sites, with neoformed collagen bundles by the delivered ASC, replacing the PLFH. There was no recipient tissue adverse reaction developed against ASC after their implantation. Pro-inflammatory cytokines (IL-1β and IL-6) gene expression confirmed the early inflammatory phase 24 h after implantation and then a strong reduction overtime back to baseline at day 30 (Figure 5D,E and Figure 6D,E). There was no difference in expression between PLFH with or without ASC, except at 24 h, where a higher level of IL-6 expression in the group with ASC was observed (Figure 5E).

### 2.7. ASC in PLFH Carrier Engraft Successfully and Persist as Substantial Fibroblastic-Like Cell Bundles

Immediately after implantation (day 1), ASC were observed as scattered (or in small clusters) non-cohesive large round cells entrapped in PLFH matrix (Figure 5A,F and Figure 6B). Death of some ASC was observed as focal areas of ghost cells or empty spaces, particularly inside or close to the calcified areas (Figure 5A and Figure 6A). ASC rapidly evolved from round (day 1) to ovoid or spindle shaped (day 7) large cells, some being observed at the PLFH periphery (Figure 5F). Dense bundles of fusiform ASC highlighted at day 18 and 30 were associated with the synthesis of extracellular collagenous matrix (Figure 5B,C). Higher magnification suggested the local mobility capacity of the cell to infiltrate the surrounding tissues. They were found as isolated or small clusters of fibroblast-like cells, preferentially in the horizontal plane of the subcutaneous connective tissue, creeping between the muscular fibers or in perivascular positions (Figure 5F). Ki-67 positive human ASC were observed inside the carrier (day 1), then outside (day 7 to 30). Their proliferative index progressively decreased from 20% (early timepoints) to 5% day 7 after implantation (Figure 5G,I). Their density increased between day 1 and 7 after PLFH implantation (Figure 5H and Figure 6C). These results demonstrated that ASC substantially integrated the recipient tissues and persisted throughout the experiment.

### 2.8. Biodistribution and Toxicity Study Suggest the Absence of Distant Distribution of ASC Entrapped in the PLFH Carrier and No Evidence of General Toxicity

PLFH mouse subcutaneous and subgingival PLFH delivery procedures (with or without ASC) did not result in any mortality nor local or systemic adverse effect. At necropsy, there was no recorded macroscopic changes. HE histological examination of the various sampled organs (drainage lymph nodes, lungs, spleen, liver and kidneys for subcutaneous graft, drainage lymph nodes and lungs for subgingival grafts) did not reveal any findings related to the ASC on this set of distant organs, ruling out a general toxicity of the locally implanted combined PLFH/ASC ATMP. The biodistribution analysis (Supplementary Figure S4) focused on locoregional drainage lymph nodes of the grafted sites and main organs that can be reached by systemic vascular dissemination (test for the worst-case situation, where some ASC would have entered the vasculature). Despite the local engraftment of a large number of ASC from day 1 to 30 at the delivery site, the immunohistochemical anti-human cell tracking in mouse tissues did not identify human ASC in the distal tissues outside PLFH/ASC grafted site at any time point, neither in locoregional (lymph nodes), nor in distant organs.

## 3. Discussion

To overcome the current limits associated with therapies based on cell suspension injection, the aim of engineered biomaterial is conversely to allow a proper tissue integration by recapitulating the physiological microenvironment, thus providing essential biophysiochemical signals that may preserve stemness, direct cell fate and differentiation, promote reprogramming by acting on genomic and epigenomic features, and drive cells towards the appropriated functional phenotypes. It is assumed that the encapsulation of MSC with hydrogels enhance their therapeutic efficacy. Such 3D scaffolding of MSC before administration seems to improve local targeted delivery, increase the retention at the injury site, and provide tunable elastic materials with many tissue-like properties essential to enhance the function of MSC, including the continuous release of the MSC secretome [29,30]. In addition, biomaterials represent active biological interfaces that may contribute to remodeling the host environment by facilitating cell recruitment and migration as well as vascularization, a decisive step in long-term cell implantation and ultimately tissue regeneration.

In order to develop a 3D hydrogel to enhance the ASC transplantation outcome, this study reported the development of a PL-based biomaterial for tissue regeneration from in vitro mechanical and biological characterization to in vivo preclinical testing. We were able to determine the combined ATMP safety profile and tolerance and the PLFH properties to support cells viability and migration into the host environment. It is assumed that carriers need to mimic the microenvironment of endogenous cells and may contribute to their cell fate by enhancing cell-extracellular matrix interactions.

We have demonstrated that an accurate control of PL and $CaCl_{2}$ amounts can modify pore width, fibrin fiber density and size and polymerization kinetics. Optical density monitoring showed the polymerization after an average of 20 min, which contrasts with reports of a turbidity increase immediately after mixing the constituents [31]. This could be explained by the different nature of the blood derivate products used: PRP [31], whose platelets are unimpaired and whose coagulation cascade can be reproduced, versus platelet–lysate, whose platelets have been previously disrupted by heat shock in the present study. Hydrogels were also highly porous and allowed the diffusion of dextran molecules with molecular weights close to the growth factors involved in tissue engineering [32]. SEM observation highlighted different fibrin fibers sizes, as a network similar to that already reported [33,34]. Increasing the amount of CaCl2 was related to slower polymerization and obtaining smaller fibers in a consistent manner with previous reports [31], impairing the mechanical qualities of the hydrogel. The final hydrogel product was malleable enough to fill a tissue defect (subgingival site) and strong enough to retain its shape over time despite progressive degradation (subcutaneous implantation).

The use of PL as a biomaterial provides many advantages for MSC therapy. In a coagulated form, PL organizes as a hydrogel of natural fibrin, combining ergonomic handling, adequate wound filling, functional support for grafted MSC and microenvironment conditioning close to the endogenous context. Indeed, the three-dimensional fibrin network mimics an extracellular matrix [22], providing a bioactive structure, which facilitates graft colonization by vessels and supports retention and prolonged delivery of growth factors (from PL *per se*, paracrine activity of ASC and host growth factors) [22]. PL contains hundreds of bioactive proteins [35] that can explain both the beneficial effects of the lysate in repair processes (helping for chronic wound healing [36], including chronic skin ulcers and oral diseases) and to provide a supportive physico-chemical environment to ASC. To that end, fibrin hydrogels are reported to display a great biocompatibility with ASC supporting angiogenic behaviour [37,38,39]. Growth factors are also found in other platelet concentrates such as PRF and PRP, which are already routinely used for skin/gingival wound healing and tissue repair [11,40]. However, the main difference between platelet concentrates and PL relies on growth factors’ availability and release, since growth factors are all liberated into the PL and captured within the hydrogel for delayed release.

During polymerization, a dense fibrin network trapped the cells. From 72 h, ASC showed spreading and adhesion within the matrix network, without migration capacity failure. The embedding process did not alter cell viability except for gel 3 (lowest amount of PL), where preliminary observations by optical microscopy displayed a higher cell mortality compared to the other hydrogels. These results support the importance of the cell–matrix link to support cell survival [41]. Indeed, a fibrin network supporting cell–matrix interactions may be of decisive importance for mechano-transduction and cell signaling. Additionally, it is reported that MSC can degrade the fibrin network, a key role in cell migration and invasion as well as for matrix remodeling [42]. ASC proliferated over time with average doubling time in PLFH (4.4 days) similar to the control cells growing in 2D culture. PL are successfully used for culturing MSC intended for human cell therapy (bone marrow [43,44], umbilical cord [45,46], adipose tissue [47,48,49], muscle [50], amniotic fluid [51], etc.). It promotes the metabolic activity, supports ASC proliferation [44,52,53] and differentiation potential [54,55,56,57,58,59] without chromosomal aberrations [60,61]. ASC senescence is reduced when cells were cultured in the presence of PL rather than bovine serum [62].

Use of PL is compatible with the addition of other biomaterials, which could open up other fields of application and additional properties, such as collagen [63], hyaluronic acid [64], PLGA (for poly(lactic-co-glycolic acid) [65,66], polycaprolactone [67,68,69] or even alginate-chitosan chronic inflammatory conditions, for example on skin and tissue wounds [70,71] or even mucositis [72]. In the latter case, seven patients were treated for gingival inflammation with a platelet lysate-based mucoadhesive formulation, and six of them showed a favorable response to treatment. Nevertheless, with a view to standardization for the translation to human, safe blood-derived products should be available in sufficient quantity and with reproducible protein content across donor pools [70,71]; it is therefore important to use pooled products to smooth the inter-donor variability. Developing functionalized hydrogels through the combination of biomaterials may also allow one to have better control matrix stiffness and biodegradability.

The purpose of the current study was also to evaluate the in vivo PLFH implantation feasibility, local evolution of both the carrier and the cells and to provide biodistribution and safety data in two different local delivery sites (subcutaneous and subgingival).

Only a few ATMP have received a European Union market authorization, and this rate of new product authorization is considered low compared to the authorization rates of other types of medicinal products [73]. For cell therapy products, i.e., the human ASC component of the present ATMP, the common recommendations of European and American medical agencies (FDA (Food and Drug Administration) and EMA) along with the ISSCR (International Society for Stem Cell Research) ask for safety and efficacy as the purpose of preclinical biodistribution studies [74]. Among others, they recommend an estimation of the duration of cell survival, assessment of the rationale for the administration method (delivery route), the distribution of the cells after administration and engraftment in ectopic sites. FDA and EMA also recommend biodistribution assessment for the evaluation of the state and function of the cells (proliferation, differentiation and migration) [74,75].

Well designed Good Laboratory Practice (GLP) compliant safety studies with sufficient large group sizes and long observation times are essential before clinical trial. However, this non-GLP preclinical study was conducted with regards to the regulatory recommendations in order to give critical preliminary results of the biosafety profile that could support the design of further studies for the translation to human [75]. In this regulatory context (EMA/FDA guidelines), animal models are essential to assess biological activity and mandatory for non-clinical safety studies [76]. The animal species tested must be the most suitable for the determination of cell fate, delivery and dosing through the biodistribution studies. The choice of the nude model in this xenogeneic design was to preserve the inflammatory response while avoiding the putative cell rejection related to the adaptive immune system. In humans, MSC have the potential to survive for several days in the recipient, through their host immune tolerance (low expression of the Major Histocompatibility Complex—MHC [77]).

The optimal cell dose for a given delivery route is also a question to be addressed [78,79]. In the present study, the ASC dose was chosen based on preliminary data to maximize cell number and viability, while remaining compatible with translation to human [80]. Despite some ASC death, possibly due to the calcium salt precipitation, a large number of cells survived, proliferated and engrafted, suggesting that a single administration should be efficient to reach a therapeutic efficacy. The observation of dense differentiated human fibroblast such as bundles firmly implanted, with low but significant proliferative index at late time points, may suggest a satisfactory ASC dose and cell/carrier ratio in the ATMP. One of the corollaries of the successful and stable engraftment of ASC is the need to further consider the tumorigenicity risk in the safety profile. Tumorigenicity assessment requires a longer term (3 to 6 month duration) in vivo grafting experiments and may be required for further translation to humans (even if this risk is very low [81]).

The precise identification and tissue tracking of post-delivery cell products is a technological critical issue that cannot be circumvented to access to a better understanding of biology and the fate of transplanted cells and that must meet the requirements of the medical agencies and the ISSCR recommendations (reliable biomarker for long term follow-up and high sensitivity method) [82]. We used here a simple, accurate, and sensitive method on murine tissues, the specific anti-human vimentin. HE slides evaluation and IHC labelling allowed one to evaluate the tissue distribution after local delivery, the persistence over time, the potential engraftment in ectopic or distant sites, ASC viability, proliferation index and local migration, as well as the recipient tissue reactions (inflammatory reaction and surrounding fibrosis).

In vivo implantation of PHFL with and without ASC provides data on the biodegradability and tolerance of the combined ATMP. As expected, the fibrin content of the carrier exhibited tissue biocompatibility and controlled biodegradation [83] The biodegradability of the carrier was demonstrated as a progressive reduction and disappearance of the supporting gel by fragmentation, degradation and precipitation of calcium salts. The development of a granulomatous foreign body giant cell inflammatory reaction against the calcium foci was observed with variable severity in most cases and with a gradual reduction. This type of chronic inflammatory reaction after the implantation of various biomaterials or medical devices is a common biological tolerance finding devoid of severity, as it tends disappear within a few weeks [84]. This transient infammatory reaction may even be considered as a conditioning signal to commit the repair process toward regeneration (instead of scarring), especially with MSC association [85,86].

Overall, we were able to demonstrate the long-term survival, engraftment and tissue integration as well as collagen matrix secretion of PLFH/ASC, suggesting that this ATMP provides an efficient physico-chemical support for the survival of ASC. Most grafted cells in the carrier certainly survived and developed a long fusiform stromal phenotype, suggesting cell–cell interaction and oriented bundles’ organisation along with collagenous extracellular matrix secretion, which also constitutes a favourable niche for persistence. Regarding the biodistribution, such a local carrier-based site is less detrimental than the general blood circulation injection followed by secondary tissue entrapment of the therapeutic cells. Local injection of ASC in suspension can lead to the mechanical drainage of cells that can passively migrate along the anatomical route to reach the drainage lymph node or the blood vessels and further the lungs and other organs, such as the liver and spleen [78,87]. Comparatively, the absence of off target tissue biodistribution of ASC using PLFH carrier highlights the capacity of the gel to completely entrap and retain the ASC to a strictly local delivery from 1 day to 1-month post-surgery in our immuno-deficient mouse model. Moreover, the absence of toxicity findings on the sampled organs combined with the absence of distant biodistribution lower the need of a general toxicity study before the translation to human.

## 4. Methods and Materials

### 4.1. PLFH Preparation

Human PL have been obtained from batches prepared by the French Blood Establishment (i.e., EFS). PL were then mixed with an activating solution containing calcium chloride ($CaCl_{2}$ 10% Solution Injectable, Renaudin, Itxassou, France), sodium chloride (NaCl 0.9% Solution Injectable, PROAMP, Aguettant, Lyon, France) and tranexamic acid (TA Exacyl 0.5 g/5 mL, Cheplapharm, Levallois-Perret, France). FH-PL were made at room temperature (RT) in 24-well plates in 3 layers of 500 µL per well, except when notified. Once constituted, PLFH were kept in a 37 °C for their polymerization during various times, as detailed below.

### 4.2. PLFH Polymerization Assessment

Determination of the impact of the component (PL, NaCl, $CaCl_{2}$ and TA) proportion on PLFH polymerization probability and rate was performed using a “mixture experiment” methodology [88]. PLFH polymerization was monitored by 640 nm optical density assessment over time (1 h, 37 °C) (Varioskan, ThermoFisher Scientific, Illkirch, France). The polymerization time is defined as the time at the intersection of the tangent to the exponential growth phase with the asymptote (Figure 2A).

### 4.3. Rheology

A 20 mm diameter grid and plate rheometer (Haake MARS 40, ThermoFisher Scientific, Illkirch, France) was used to measure the viscoelastic properties of PLFH (the temperature was set at 20 °C for these experiments). The 4 mL hydrogels used in this experiment were formulated as described above with a 0.45 µm-filtered platelet lysate. Strain sweeps from 0.1 to 100% strain at 1 Hz were performed to define the linear regime and then frequency sweeps from 1 Hz to 0.1 Hz at 0.5% strain were performed to determine the frequency-dependence of both the storage modulus (G’) and the loss modulus (G”). The average moduli over the frequency range were calculated for the four PLFH conditions.

### 4.4. Surface Resistance of the Hydrogel

The surface behavior of 1 mL hydrogel (9.5 mm diameter, 10.5 mm height, contained in cylindrical glass containers) was studied using a texturometer (TA.XT Plus Texture Analyzer, Stable Micro Systems, Godalming GU7 1YL, UK) set in compression with a 4 mm diameter cylinder stainless probe at a crosshead speed of 2 mm per minute. The recording was stopped after the rupture of the gel’s surface layer, and the maximum stress applied was thus noted.

### 4.5. Electron Microscopy

PLFH was recovered from the wells and its central part was cut with a blade as a 1 mm${}^{3}$ piece. To avoid deformation, the samples were immediately frozen under high pressure (EM ICE, Leica, Nanterre, France) and stored in liquid nitrogen. The samples were subsequently observed by scanning electron cryomicroscopy (Quanta 250 FEG FEI, ThermoFisher Scientific, Illkirch, France) after a sublimation time of 30 min and platinum deposition (PP3000T, Durom).

### 4.6. PLFH Porosity Assessment (dextran)

Porosity was assessed by monitoring the diffusion of high molecular weight dextran through the fibrin network [89]. A 250 µL solution at 0.5 mg/mL of 70 kDa fluorescein-dextran (Sigma-Aldrich, Saint-Quentin-Fallavier, France) was deposited on top of the gel, and the supernatant was collected at 24 h. Fluorescence was measured at absorption/emission wavelengths at 496/520 nm (Varioskan, ThermoFisher Scientific, Illkirch, France).

### 4.7. PLFH Swelling and pH Assays

PLFH swelling corresponds to its hydrous imbibition. The gels were collected and weighed on a precision balance. They were then dried on a compress and reweighed. The swelling corresponds to the difference between the weight of the wet gel and its dry weight, expressed in absolute values. PLFH pH was assessed with an electronic pH meter (WTW Bioblock, ThermoFisher Scientific, Illkirch, France) at room temperature immediately after their constitution.

### 4.8. Human ASC Culture and Incorporation in PLFH

ASC were isolated from human subcutaneous adipose tissue samples following a previously described protocol [90]. Briefly, tissue was digested for 45 min at 37 °C in αMEM + 2% PL medium and 0.8 U/mL collagenase type NB4 (Sigma-Aldrich, Saint-Quentin-Fallavier, France). The digestion was then 100 µm-filtered and centrifuged at 360× *g* for 8 min to remove debris and mature adipocytes. Red blood cells were lysed in hypotonic buffer containing 140 mM $NH_{4}Cl$ and 20 mM Tris for 5 min at 4 °C. The vascular stromal fraction was further recovered by centrifugation at 290× *g* for 5 min. After counting, cells were seeded for expansion at 4000 cells/cm² in PL αMEM + 2% PL medium containing 0.25 mg/mL amphotericin B, 100 mg/mL streptomycin, 100 IU/mL penicillin, and 5 IU heparin per PL mL until reaching confluency, then passaged. For PLFH/ASC constitution, cells from passage 1 or 2 were used during the mixture polymerization. ASC culture and flow cytometry characterisation are presented in Supplementary Figure S4.

### 4.9. Cell Viability

PLFH were performed with 200,000 ASC per layer, then kept at 37 °C in αMEM + 2% PL. After 24 h, PLFH/ASC were rinsed with PBS then incubated at RT for 90 min with 1/5000 DAPI and 1/500 CFDA solutions (labelling dead and live cells, respectively). PLFH/ASC were washed with PBS (two 10-min washes) then imaged with Operetta® (Perkin-Elmer, Villebon-sur-Yvette, France ) to assess cell viability.

### 4.10. DNA Quantification

For DNA assessment over time, 20,000 ASC per layer were seeded in PLFH, kept at 37 °C in αMEM + 2% PL, then PLFH/ASC were rinsed then frozen at −80 °C in RLT buffer (Qiagen, Courtaboeuf, France ) 3 h later (day 0) or at day 1, 3, 5 and 7. Samples were lysed with a bead on tissue lyser (2 × 2 min at 20 beats per second) and then centrifuged (15 min, 16,000× *g*, 4 °C). The Quant-iT PicoGreen dsDNA Assay kit (ThermoFisher Scientific, Illkirch, France ) was used for quantification, according to the manufacturer’s recommendations. The results were adjusted to the basal DNA level of the cell-free hydrogel. Cells left adherent on plastic were used as control.

### 4.11. In Vitro Ki-67 Immunofluorescence

For the Ki-67 immunofluorescence assay, 100,000 ASC were seeded in PLFH in 8-wells Labtek (Sigma-Aldrich) then kept in αMEM + 2% PL for 72 h followed by PFA 3.7% fixation. Samples were then incubated in 10% normal goat serum for 2 h at room temperature. Rabbit anti-Ki67 (1:250) and CFDA (1:500) were then added overnight at 4 °C. After washing, samples were incubated with Alexa 555 donkey anti-rabbit (1/500) (Merck) for 1 h at RT before 1/5000 DAPI staining, then washing. Primary antibody was omitted in negative controls. Images were acquired with confocal LSM 710 microscope (Carl Zeiss, Rueil-Malmaison, France ).

### 4.12. Cell Migration

A total of 200,000 ASC per layer were seeded in PLFH and kept at 37 °C in αMEM + 2% PL for 72 h. Then, PLFH/ASC were removed from wells and dropped as explants in 6-well plates. After 5 days, the wells were fixed with 3.7% PFA and stained with May-Grünwald Giemsa (RAL stainer kit MCDh, RAL Diagnostics, Bordeaux, France). The cell release and migration were quantified by measuring the distance (in µm) between the gel and the cell migration front.

### 4.13. In Vivo Experiment

All animal experiments were carried out by specialized researchers, engineers and technicians, meeting the requirements of the protection of animals used for scientific purposes, as defined in the European regulation (directive 2010/63/UE) and the applicable French welfare legislations. All animal experimentation procedures were approved by the competent authorities (MESR- APAFIS n°16348-2018073110473357v3 and 22399-2019100916269812v3). Mice were bred in the CREFRE facility (Centre Regional d’Exploration Fonctionnelle et Ressources Expérimentales) and maintained in microisolators and ventilated racks to reduce potential exposure to pathogens. Mice were housed in a controlled environment (12 h light-dark cycle, 22 °C, 60% humidity), fed ad libitum with complete diet and had free access to water. At the initiation of treatment, the animals were 7–8 weeks old. For ethical considerations, we performed a combined study of biosafety and biodistribution of the local surgical graft of the PLFH.

PLFH with or without ASC were grafted in immunocompromised mice (Athym-Foxn1 nu/nu males from Janvier Labs, Le Genest-Saint-Isle, France) to overcome the host immune response to human cell transplantation. Using 200,000 ASC per PLFH layer, the cell concentration was therefore set to $0.4\times 10^{6}$/mL as defined by Pers et al. [80]. PLFH with ASC (experimental group) or PLFH without ASC (control group) were implanted subcutaneously in mice, at the flank level (1 per side) by a simple median skin incision and flap removal of adherent tissue planes over a small area corresponding to the volume of the biomaterial under 2–3% isoflurane in a 50/50 air/$O_{2}$ mixture. For oral study, a miniaturization of the graft was performed in a 96-well plate with 3 layers of 100 µL (the same ASC ratio as subcutaneous implantation assays). A lingual gingival flap was made under binocular microscopy in the mandibular first molar region. Then, PLFH was slipped underneath the gingiva in each of the right and left mandibular sectors of the mouse. No suture was required, as the flap repositioned spontaneously. PLFH consistency and elasticity were suitable to be handled during surgery and retained both in subcutaneous and subgingival sites. The toxicity was assessed during the *in-life* phase on survival, welfare, clinical signs, and by the macroscopic changes recorded at necropsy. At different time points (24 h, 72 h, 7, 18, and 30 days), animals (6 to 9 mice at each timepoint) were euthanized.

Subcutaneous PLFH/ASC implanted on the left side of the animal was used for histological study, while the right-side one was recovered using an 8 mm biopsy punch for RT-qPCR transcriptional analysis. To assess the systemic biodistribution and toxicity, axillary, inguinal, and iliac lymph nodes as well as kidneys, livers, spleens and lungs were sampled. Samples in the oral position were collected 24 h, 72 h and 7 days after implantation (3 mice at each time). Similarly, the left hemi-mandible was devoted for histology and the right for RT-qPCR (for the latter, sampling was restricted to the molar sector, corresponding to the site of the graft). For biodistribution, cervical lymph nodes and lungs were sampled. Samples for histology were fixed in 10% buffered formalin for 2 days. Samples for RT-qPCR were stored at 4 °C for 24 h in RNA-later and then frozen at −80 °C until processing.

### 4.14. Histology and Immunohistochemistry

Hemi-mandible specimens were decalcified in 10% EDTA at 37 °C for 7 days before routine processing. Fixed tissues and organs were embedded in paraffin wax, sectioned in 3 µm-thick slices and processed for hematoxylin and eosin and Masson trichrome stainings. Anti-human-vimentin immunohistochemistry (IHC) was used as a specific, accurate and sensitive method to track the transplanted human ASC [74], and anti-human Ki-67 IHC to estimate their proliferation. Staining of 3-µm sections of paraffin-embedded specimens was performed after antigen retrieval for 30 min (Ptlink, low pH, ref K8005, Dako, Les Ulis, France), using anti-vimentin antibody (M0725, mouse monoclonal antibody, Dako, Les Ulis, France, dilution 1/50, 50 min at RT) and the ARK (Animal Research Kit, Dako, Les Ulis, France) or anti-Ki-67 antibody (ref K4061, mouse monoclonal antibody, Dako, Les Ulis, France, dilution 1/50, 50 min at RT). Staining was carried out with Dakostainer automated system using the envision secondary step and DAB as chromogen. Proliferation index (number of Ki-67 positive ASC on total cells) and cell density (number of vimentin positive ASC on total cells) were assessed on scanned slides (Scanner Pannoramics 250, 3DHistec, Brignais, France) and analyzed by manual counting on the region of interest with a panoramic viewer.

### 4.15. RTqPCR

Skin or gingival samples were mechanically disrupted with a steel ball (“tissue lyser”). After the addition of chloroform and centrifugation (15 min, 16,000× *g*, 4 °C), the aqueous phase containing nucleic acids was collected. The RNeasy Micro Kit (Qiagen, Courtaboeuf, France) was used for RNA extraction according to the manufacturer’s protocol. The assay of these RNA was performed with Nanodrop 2000c (ThermoFisher Scientific, Illkirch, France) at 260 nm and then the synthesis of complementary DNA (cDNA) was performed from 1000ng of RNA using the high-capacity cDNA reverse-transcriptase kit according to the manufacturer’s recommendations (Applied Biosystems, Illkirch, France). RT-qPCR was performed on StepOne (Applied Biosystems, Illkirch, France) using the Fast SYBR Green MasterMix (ThermoFisher Scientific, Illkirch, France) to evaluate the gene transcription at different times encoding the proteins IL-1β (F: 5’CCACAGACCTTCCAGGAGAAT’, R: 5’GTGCAGTTCAGTGATCGTACA’) and IL-6 (F: 5’ACACATGTTCTCTGGGAAATCGT’, R: 5’CAAGTGCATCATCGTTGTTCATAC’). Gene expression was quantified by the Ct (threshold cycle) method using the Ct of the 18S (F: 5’AGTGGGGGACTAGGCGTTGTTAG3’, R: 5’GTTTTCATCACTACTGTCTGCATCC3’) and 36B4 (F: 5’GTCTCTGCCGCCCTTCTGTGC’, R: 5’CAGCAGGTGACTGGGGCATTG’) gene as reference.

### 4.16. Statistical Analyses

The comparison of the different hydrogel conditions was performed by the analysis of variance (ANOVA) at the 5% significance level, taking into account multiple comparisons (Bonferroni correction). Stata v13®, R® and GraphPad Prism 5® software were used for data processing. For the mixture experiment, the R packages “mixexp” and “AlgDesign” were used to define the mixture values to be tested and to model the probabilities and the polymerization times. Each experiment was replicated at least 5 times. Data were expressed as mean ± the standard error of the mean unless specified.

## 5. Conclusions

Taken together, our data strongly support the safety of our ATMP for a human clinical purpose. Indeed, the PLFH accurate design development, biomechanical property determination, cell activity and safety demonstration provide robust prerequisite to go further in the translational research and clinical trials in humans. These encouraging results are the basis for regulatory preclinical testing for the Investigational Medicinal Product Dossier.

## Data Availability

Data is contained within the article or supplementary material.

## Supplementary Materials

The following supporting information can be downloaded at: https://www.mdpi.com/article/10.3390/ijms24032206/s1.
