# Supplementary material for: Characterization and Safety Profile of a New Combined Advanced Therapeutic Medical Product Platelet Lysate-Based Fibrin Hydrogel for Mesenchymal Stromal Cell Local Delivery in Regenerative Medicine
Source: Int J Mol Sci. 2023 Jan 22;24(3):2206. doi: 10.3390/ijms24032206 (PMC9916739; doi:10.3390/ijms24032206)
Supplement: Supplementary file 1 [file ijms-24-02206-s001.zip › ijms-2144430-supplementary.pdf]

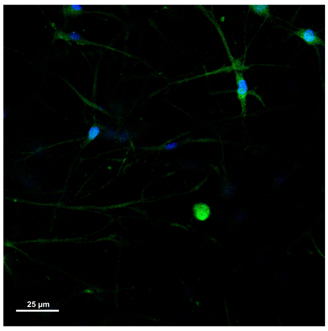

Supplementary Figure S1: Ki-67 immunofluorescence negative control, where primary antibody was omitted.

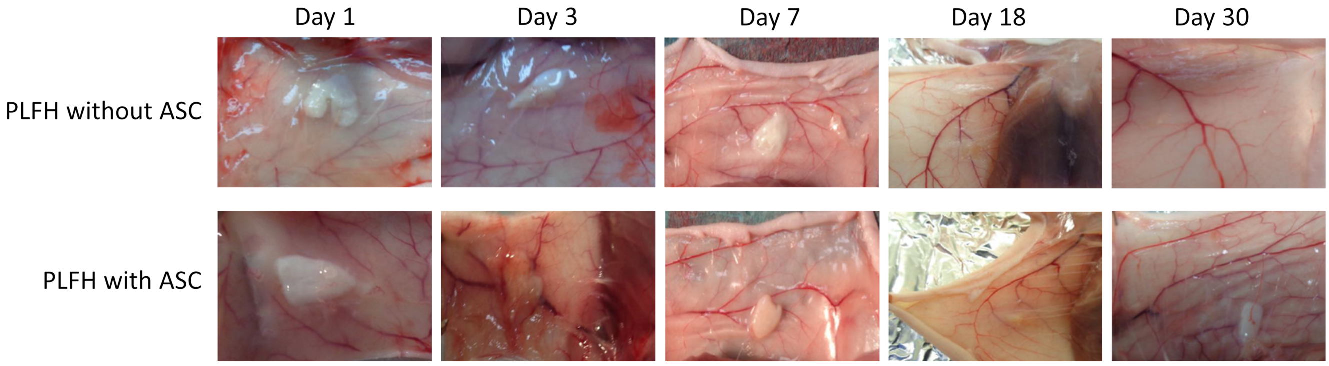

Supplementary Figure S2: Macroscopic observations. Most representative acquisitions of the graft areas after sacrifice, with localization of the biohybrid after skin detachment at different kinetics. During the early kinetics (day 1 to day 7), PLFH were easily identifiable through the skin of the animals. After lifting the flap, a similar appearance was found between the two groups at day 1 and day 3, with an oval shape. After 7 days, the cell-free PLFH had a more pronounced pearly white color. At late kinetics (day 18 and day 30), PLFH were more difficult to recognize, being absent or in small fragments.

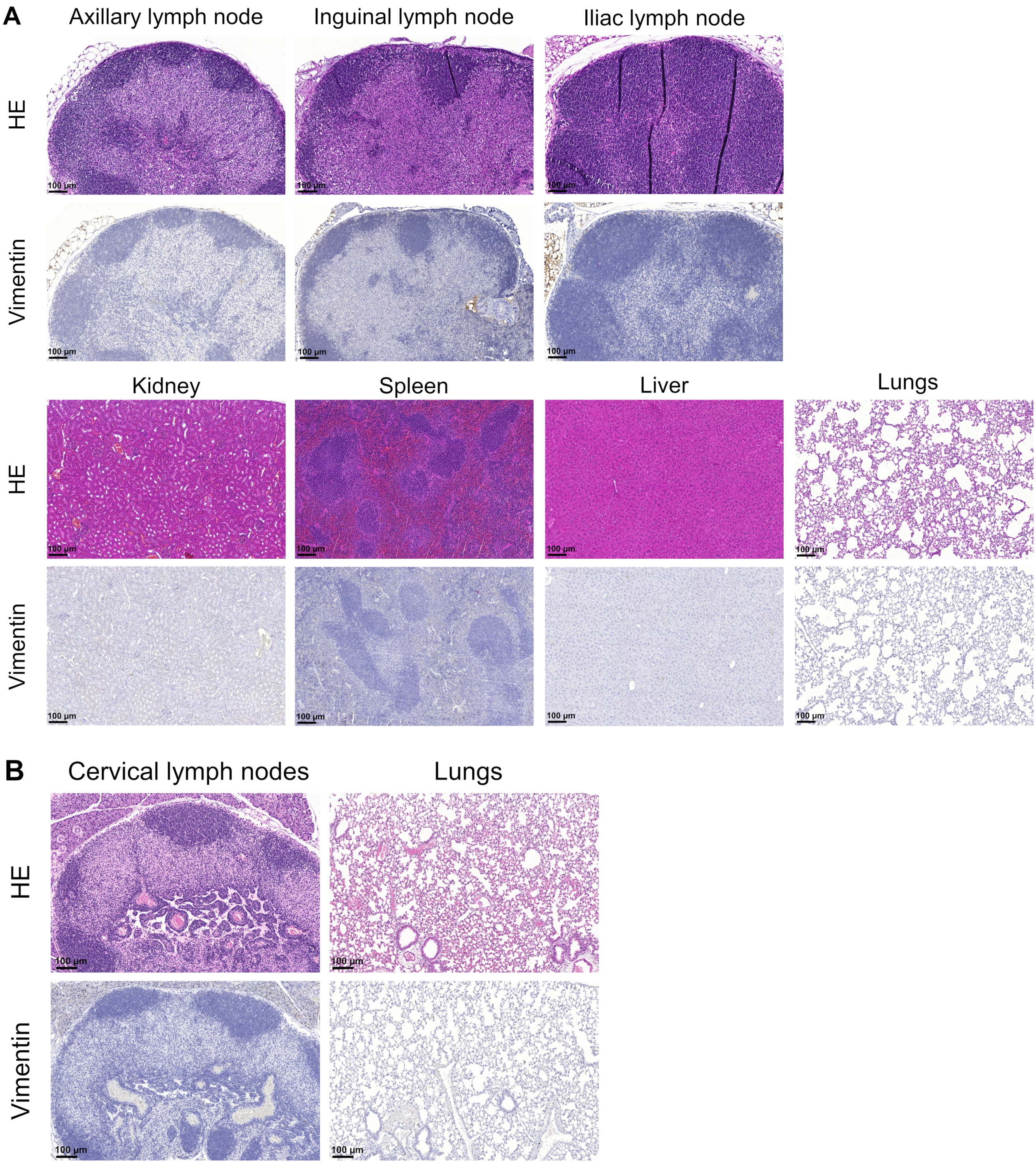

Supplementary Figure S3: Combined biodistribution and toxicity study. Histopathological evaluation of Hematoxilin-Eosin stained slides on a set of sampled tissues (locoregional drainage lymph nodes and distant organs) for each timepoint (day 1, 3, 7, 18 and 30) did not demonstrate any microscopic changes related to the PLFH/ASC ATMP subcutaneous (A) or subgingival (B) implantation for toxicologic purposes (HE staining, bar = 100µm). Microscopic tracking of vimentin positive human ASC in the same set of tissues did not allow to identify human ASC in the drainage lymph nodes and the distant organs (Vimentine IHC, bar = 100µm) for biodistribution study. Representative illustrations for subcutaneous and subgingival grafts are from day 1.

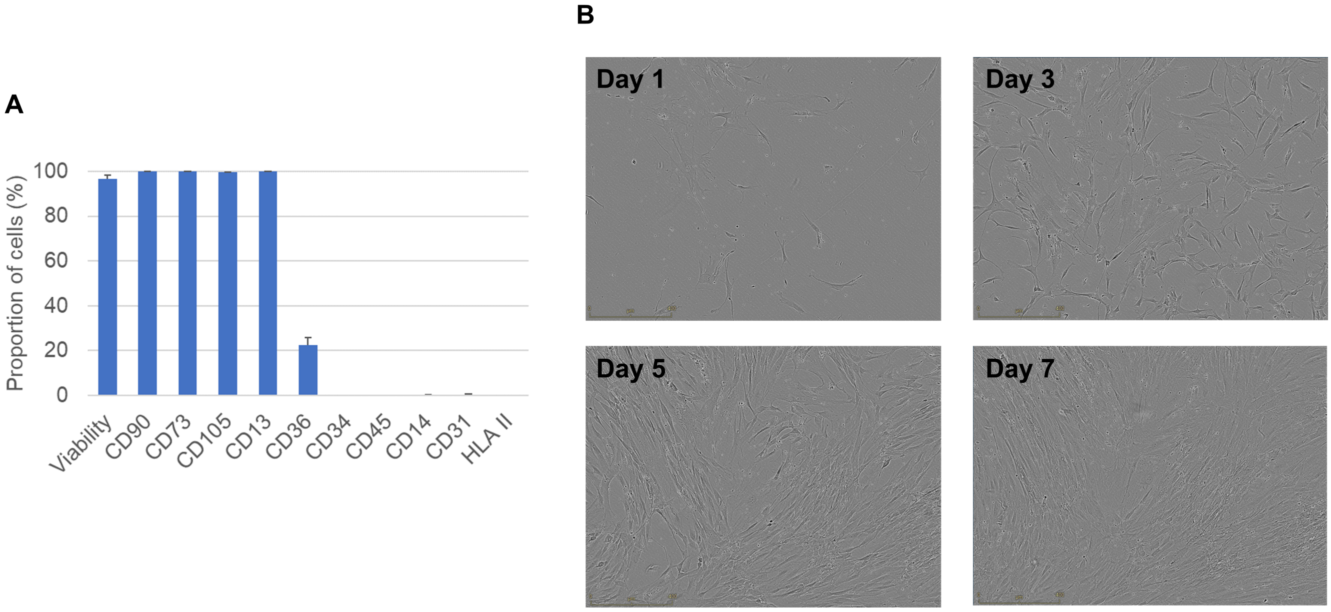

Supplementary Figure S4: ASC caracterisation by flow cytometry (A). Photographs of ASC during culture (B). Bar scale 400µm.
